# A systematic study on drug-response associated genes using baseline gene expressions of the Cancer Cell Line Encyclopedia

**DOI:** 10.1038/srep22811

**Published:** 2016-03-10

**Authors:** Xiaoming Liu, Jiasheng Yang, Yi Zhang, Yun Fang, Fayou Wang, Jun Wang, Xiaoqi Zheng, Jialiang Yang

**Affiliations:** 1 Department of Mathematics, Shanghai Normal University, Shanghai 200234, P. R. China; 2 Department of Civil and Environmental Engineering, National University of Singapore, Singapore 117576, Singapore; 3 Department of Mathematics, Hebei University of Science and Technology, Shijiazhuang, Hebei 050018, P. R. China; 4 Department of Genetics and Genomic Sciences, Icahn School of Medicine at Mount Sinai, New York, NY 10029, USA

## Abstract

We have studied drug-response associated (DRA) gene expressions by applying a systems biology framework to the Cancer Cell Line Encyclopedia data. More than 4,000 genes are inferred to be DRA for at least one drug, while the number of DRA genes for each drug varies dramatically from almost 0 to 1,226. Functional enrichment analysis shows that the DRA genes are significantly enriched in genes associated with cell cycle and plasma membrane. Moreover, there might be two patterns of DRA genes between genders. There are significantly shared DRA genes between male and female for most drugs, while very little DRA genes tend to be shared between the two genders for a few drugs targeting sex-specific cancers (e.g., PD-0332991 for breast cancer and ovarian cancer). Our analyses also show substantial difference for DRA genes between young and old samples, suggesting the necessity of considering the age effects for personalized medicine in cancers. Lastly, differential module and key driver analyses confirm cell cycle related modules as top differential ones for drug sensitivity. The analyses also reveal the role of *TSPO*, *TP53*, and many other immune or cell cycle related genes as important key drivers for DRA network modules. These key drivers provide new drug targets to improve the sensitivity of cancer therapy.

Predicting drug response of a patient based on various genetic information is a fundamental problem in current research of precision medicine. It is known that drug metabolism varies among patients, and some patients will respond faster to drugs than others^1^. Drug sensitivity is a lower threshold to achieve the normal pharmacological action of a drug^2^. Accurate prediction of drug response is very important for disease therapy and safety of patients. However, the biological mechanisms underlying the heterogeneity of individual drug response remain elusive. Recent studies have suggested that various cancer genomic markers are highly associated with anti-cancer drug sensitivity, and patients have been benefited from the drugs related to these biomarkers in clinical trials. For example, the usage of drugs targeting drug response-associated (DRA) fusion gene *BCR-ABL* in chronic myeloid leukemia^3^ and gene *BRAF* in melanoma^4^ have substantially improved the survival rate of patients. Thus, identification of DRA signatures has become an important task in personalized medicine development.

With the advent of multiple high throughput technologies, it is now practical to measure the panomics (including transcriptome, metabolome, epigenome, etc.) at a reasonable cost^5^. The rich information in panomic data provides enormous opportunities to systematically identify DRA biomarkers. For example, expressions of ATP binding cassette transporter (ABC) genes are found to be highly correlated with the response of cytotoxic drugs in cancer cell lines through an analysis of 48 known ABC transporters in 60 diverse cancer cell lines with the treatment of 1,429 anti-cancer drugs^6^. Garnett *et al.* performed a systematic analysis on 639 human tumor cell lines treated with 130 anti-cancer drugs, and identified several DRA biomarkers (e.g., fusion gene *EWS-FLI1*) as PARP inhibitors^7^. Barretina *et al.* proposed an Elastic-Net model to select anti-cancer DRA markers including gene mutation, copy number variation, and gene expression and built a drug-response prediction model using the selected biomarkers^8^.

However, cancer drug response mechanism is a very complex system that could be affected by many factors. Sex, in particular, can influence how the body handles a drug as well as the drug dose appropriate to the body^9^. Future clinical studies with patients using opioids for chronic pain should also include age as an important variable when assessing development of opioid tolerance^10^. Age is another important factor for the efficacy of drugs. It is known that there are more adverse drug reactions in the elderly than in the young, which might relate to the functional decline of clearing organs like kidney with age^11^. However, the specific genes and pathways involved in this process are not fully resolved.

Moreover, the DRA biomarkers may not function alone. Thus, it is of fundamental importance to identify not only individual gene markers, but also gene-gene interactions and modules associated with drug responses. For example, Chang *et al.* showed that pathway modules related to Ras-signaling and E2F transcription factors can be used to predict drug sensitivity^12^. By gene set enrichment analysis, it is also feasible to identify the key drivers or hub genes of a set of DRA genes in a regulatory or protein interaction network, the alternation of which will have substantial influence on the DRA gene set^13^. A number of network module methods have been developed and successfully applied in identifying the co-expression modules and key driver genes related to some diseases including Alzheimer’s disease^13^, brain cancer^14^, and so on. However, to our best knowledge, this kind of analysis to drug sensitivity is till in its infancy.

In this paper, we developed a systems biology framework to identify gene expressions, co-expressions, and co-expression modules differentially changed with drug sensitivity. We then applied this framework to the Cancer Cell Line Encyclopedia (CCLE)^7^ gene expression and drug response data and identified various DRA associated genes and modules. Moreover, we studied the effects of gender and age on DRA genes, and performed key driver analysis (KDA)^13^ on differential functional modules (with drug sensitivity) defined by Gene ontology (GO) terms and Kyoto Encyclopedia of Genes and Genomes (KEGG) pathways to identify key functional genes in the modules related to drug sensitivity.

## Results

### The CCLE data

**Figure 1:**
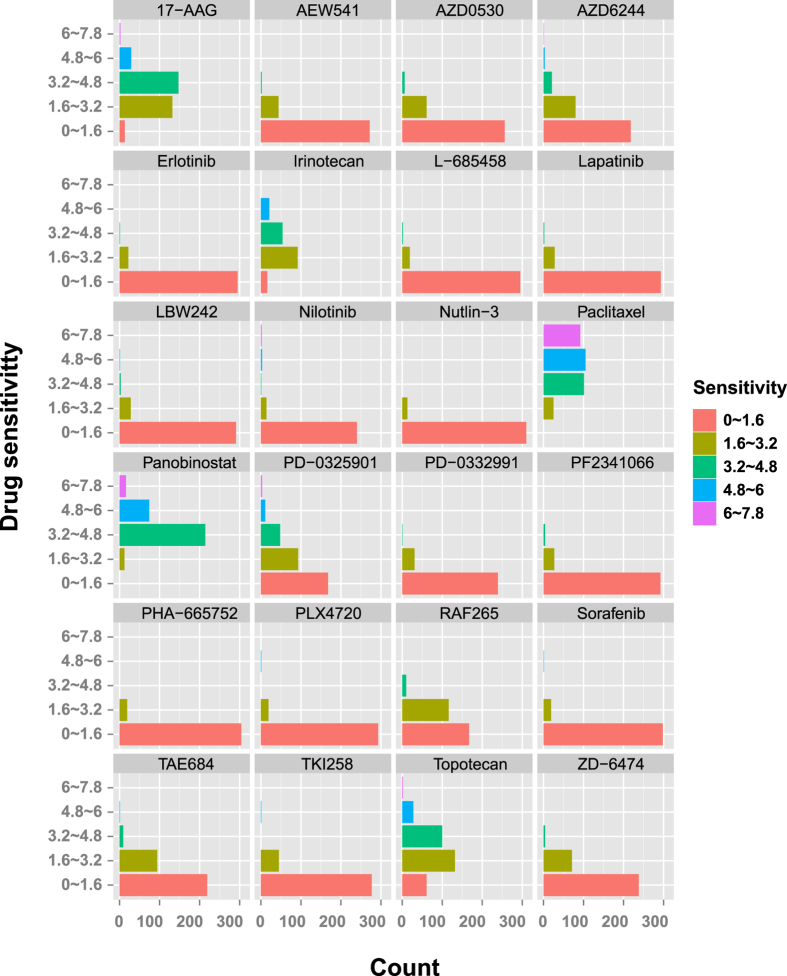
Drug sensitivity distribution of 24 drugs. Each histogram denotes the distribution of sensitivity values of a drug treating on cancer cell lines.

The CCLE project is an effort to conduct a detailed genetic characterization of a large panel of human cancer cell lines^8^. It provides baseline gene expression profile of 20,069 genes for 504 human cancer cell lines collected from 24 tissue types and 21 cancer types. These cell lines are treated by 24 anti-cancer drugs including 17-AAG, AEW541, AZD0530, AZD6244, Erlotinib, Irinotecan, L-685458, LBW242, Lapatinib, Nilotinib, Nutlin-3, PD-0325901, PD-0332991, PF2341066, PHA-665752, PLX4720, Paclitaxel, Panobinostat, RAF265, Sorafenib, TAE684, TKI258, Topotecan, and ZD-6474. It is of note that very few cancer cell lines are present for some cancer types and tissues (e.g., bone and osteosarcoma), which might lead to biased conclusion. Thus, we removed cancer types and tissues with less than 5 cell lines. Finally, we obtained 323 cancer cell lines related to 17 tissues and 7 cancers. We plotted the distribution of sensitivity values for the 24 drugs on these cell lines in Fig. 1. For a cell line, the sensitivity of a drug is measured by the area under the dose-response curves (termed as activity area)^8^, which ranges from 0 to 7.8 in this study. In addition, we collected phenotype and annotation information of the patient from whom each cell line was generated, such as gender, age, and batch, to access their impact in drug sensitivity. The detailed information can be found in Supplementary Data S1.

### Gene expression signatures of drug sensitivity

We first analyzed DRA gene expressions using a modified regression model (see Methods). Briefly, gender, age, tissue type, batch, cancer type, and top three principal components (PCs) for genotypes of cell lines are collected as potential confounding factors of drug sensitivity to cause gene expression variation. We listed the numbers of DRA genes (at false discovery rate (FDR) ≤ 0.1) for all 24 drugs in Table 1 and the detailed information for drug sensitivity association of all 20,069 genes in Supplementary Data S2.

As can be seen from Table 1, the number of DRA genes ranges from 2 to 1,226 (Lapatinib) across 24 drugs. Except for Lapatinib, there are also several hundred DRA genes identified for 17-AAG, AEW541, AZD6244, Erlotinib, Irinotecan, PD-0325901, Paclitaxel, Panobinostat, Sorafenib, TAE684, and Topotecan. Lapatinib is an orally active drug for breast cancer and other tumors^15^, and its treatment has been found to induce prevalent resistance in a couple of studies^16^,^17^,^18^. To further estimate how many false positives in our findings, we randomly permutated the sensitivity value of the cell lines for 1,000 times and counted how often we obtained more significant genes in permuted data than in the original one. In addition, we also counted the average number of significant genes for the 1,000 random permutations. The results were summarized in Table 1 under the column “Permutation”. Except for AZD0530, L-685458, LBW242, Nilotinib, PF2341066, PHA-665752, PLX4720, and TKI258, the frequencies of obtaining more DRA genes in 1,000 permutation data than in original data for other drugs are less than or equal to 1. The results suggest that most of the identified genes are truly DRA. Due to the small numbers of DRA genes (≤20) in AZD0530, L-685458, LBW242, Nutlin-3, PF2341066, PHA-665752, PLX4720, and TKI258, and high number of false positives for Nilotinib and Sorafenib (i.e., 25 and 15.36), we removed them from further study unless stated.

**Figure 2:**
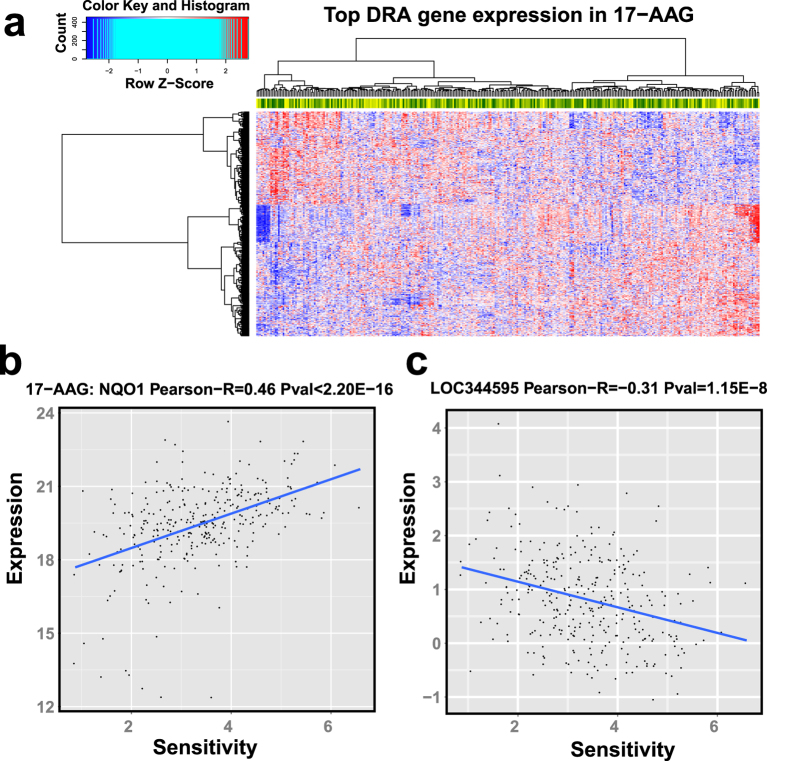
(**a**) Sensitivity-associated gene expression in 17-AAG, and scatter plot of 2 sensitivity-associated gene expression patterns, e.g. (**b**) NQO1 and (**c**) LOC344595 in 17-AAG. In (**a**), each row indicates a gene and column indicates a sample; the heat-map colors represent gene expression with red for high expression and blue for low expression. We also added a side bar at the top to indicate sensitivity value with dark green for low values and yellow for high values. In (**b**,**c**), X-axis represents sensitivity and Y-axis represents gene expression level. Pearson-R value in the title represents the Pearson correlation coefficient between gene expression and sensitivity across all samples.

As gene expression can be either positively or negatively associated with drug sensitivity and each could lead to different biological consequences, we divided DRA genes into positively and negatively associated genes (determined by the sign of the coefficient of the drug response term in the regression model). Except for Panobinostat, there are more positively associated DRA genes than negatively associated ones for other 13 drugs (Table 1). We visualized the expression pattern of DRA genes for 17-AAG in Fig. 2a and those for the other 13 drugs in Supplementary Fig. 1. As can be seen, all samples are divided into two large groups according to the hierarchical clustering (Euclidian distance with “Ward” measurement^19^). The left side group consists of more samples with relatively larger sensitivity values, and is thus referred to as “sensitive group”. The right group is referred to as “non-sensitive group”. The clear separations of “sensitive” versus “non-sensitive” group are seen in all 14 drugs. We performed the Student’s t-test between the sensitivity values of the two groups and the p-values are all smaller than 2.0 × 10^−7^ (Table 2), indicating significant difference between the two groups. In addition, we plotted the expression levels of two sample DRA genes *NQO1* and *LOC344595* for 17-AAG (Fig. 2b,c), in which each dot represents a cell line. The Pearson correlation coefficients between drug sensitivity values and gene expressions for these two genes are 0.46 (p-value < 2.2 × 10^−16^) and −0.31 (p-value = 1.2 × 10^−8^) respectively, which exhibits clear correlation between drug sensitivity and gene expression.

**Figure 3:**
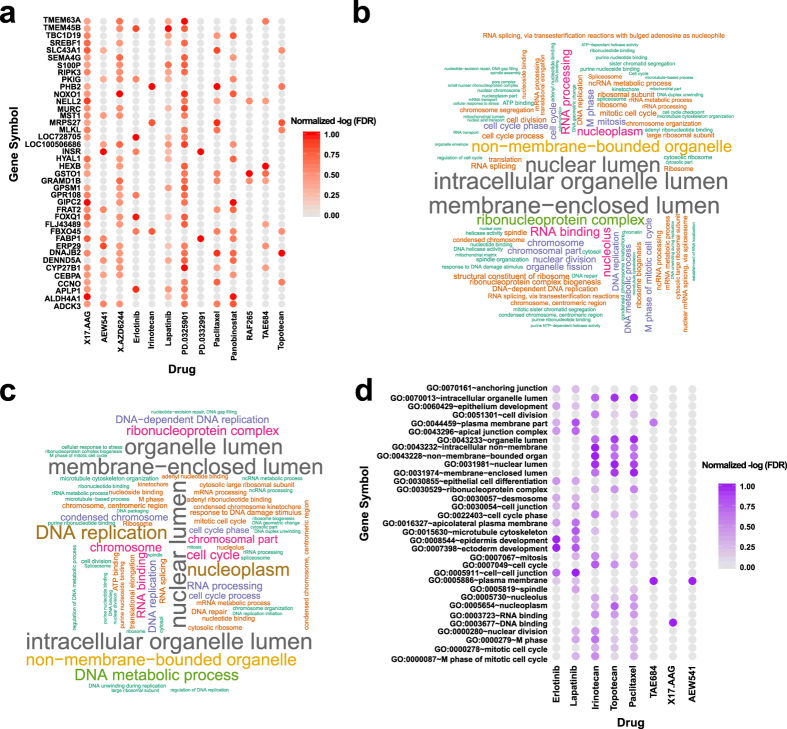
(**a**) Top sensitivity-associated genes in multiple drugs, word-plot of two drugs: (**b**) Paclitaxel and (**c**) Topotecan, and (**d**) top enriched GO terms and pathways in multiple drugs.

We next examined the overlap of DRA genes across 14 drugs. The results were summarized in Supplementary Data S3 and the top recurring genes were also plotted in Fig. 3a. Interestingly, many DRA genes occurred in multiple drugs. For example, *DNAJB2* and *ADCK* were inferred to be DRA for 6 drugs. *DNAJB2* (i.e., DnaJ (Hsp40) homolog, subfamily B, member 2) encodes a protein similar in sequence to bacterial DnaJ protein and the yeast homologs (http://www.ncbi.nlm.nih.gov/gene/3300). DnaJ proteins are differentially expressed in human tissues and demonstrate the capacity to function to both promote and suppress cancer development by acting as chaperones for tumor suppressors or oncoproteins^20^. Loss of expression for genes in this family confers resistance to chemotherapeutic agents used in treating ovarian cancer^21^. In addition, *ADCK3* gene encodes a mitochondrial protein similar to yeast ABC1, which functions in an electron-transferring membrane protein complex in the respiratory chain. Expression of this gene is induced by the tumor suppressor p53 and in response to DNA damage, and inhibiting its expression partially suppresses p53-induced apoptosis^22^. These genes might be involved in some fundamental mechanisms of multi-drug targeting process.

### DRA genes for drugs share common biological functions

The biological functions of positively and negatively associated DRA genes were annotated using David tools^23^. We plotted the word-cloud maps of the enrichment of DRA genes for two representative drugs Paclitaxel and Topotecan in Fig. 3b,c respectively and showed the enrichment of DRA genes for the rest drugs in Supplementary Data S4. The most enriched GO term for both drugs is membrane-enclosed lumen with FDR for Paclitaxel and Topotecan being 1.2 × 10^−49^ and 5.8 × 10^−23^ respectively. Membrane-enclosed lumen encompasses endoplasmic reticulum lumen, nuclear envelope lumen, and so on. A couple of studies have shown that changes in endoplasmic reticulum lumen environment affect cell sensitivity to apoptosis^24^,^25^ and are thus important for cancer treatment^26^. Other top enriched terms include GO:0007049 cell cycle with FDR being 5.1 × 10^−16^ and 2.6 × 10^−12^ and GO:0003723 RNA binding with FDRs 1.6 × 10^−22^ and 3.9 × 10^−11^ respectively for the two drugs. One of the main mechanisms of anti-cancer drugs is to induce cell apoptosis^27^. In fact, there are numerous instances of cell-cycle mediated drug resistance^28^, however the underlying mechanisms are not fully known. The DRA genes related to cell cycle might shed some light on these mechanisms. In addition, the relationship between RNA-binding and drug sensitivity (or resistance) has also been widely studied^29^,^30^.

By an overview of all drugs, we identified a few functions significantly enriched in multi-drugs. For example, GO:0005886 plasma membrane, GO:0000087 M phase of mitotic cell cycle, GO:0000279 M phase, and GO:0000280 nuclear division are significantly enriched (at FDR ≤ 0.05) in four drugs, supporting their vital roles in drug response (Fig. 3d). The relationship between plasma membrane and drug sensitivity has been widely studied. For example, several cell membrane transporter proteins including multi-drug resistance protein 1 (MDR1; also known as P-glycoprotien and ABCB1), MDR-associated protein 1 (MRP1; also known as ABCG2), and breast cancer resistance protein (BCRP; also known as ABCG2) are known to cause resistance in common chemotherapeutics by promoting drug efflux^31^. Zaman *et al.* identified that the protein MRP, which is known to be associated to multi-drug resistance, is a plasma membrane drug-efflux pump^32^. Furthermore, as the hallmark of cancer revolves around cell-cycle deregulation, it is not surprising that antimitotic therapies are effective against the abnormal proliferation of transformed cells^33^. Besides the known functions, we also identified a few less known DRA functions, e.g., spindle. The results show that drug response is a very complicated process involving a lot of biological activities.

### DRA gene patterns share certain similarity in male versus female

**Figure 4:**
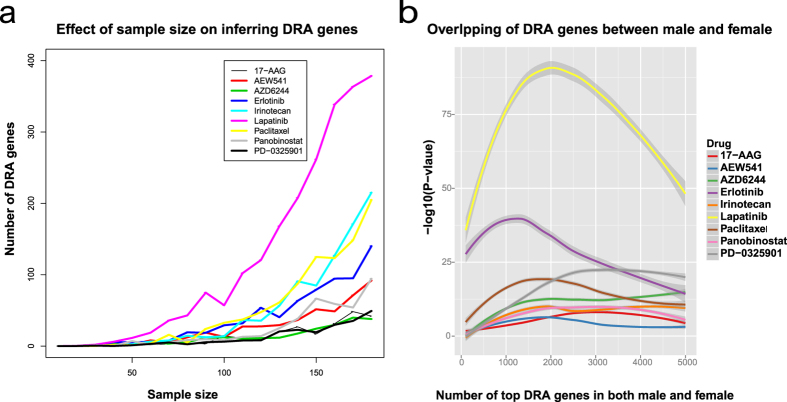
(**a**) Effect of sample size on inferring DRA genes (**b**) Fisher’s exact test on overlapping between male and female sensitivity-associated genes. In (**a**), X-axis indicates the number of top sensitivity-associated genes selected in both male and female samples. Y-axis indicates the significance of the overlap calculated based on the Fisher’s exact test.

As the sample sizes of male and females are quite different (Supplementary Table 1), we first tested the effect of sample size on detecting DRA genes. Specifically, we randomly selected 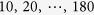 cell lines (each for 100 times) and calculated the average number of DRA genes for each sample size using the modified regression model (see Methods). The results for 9 drugs were shown in Fig. 4a and the full results for 14 drugs were summarized in Supplementary Data S5. As can be seen, the detection power varies significantly with sample size for most drugs. Thus, instead of directly comparing the number of significant DRA genes, we performed the Fisher’s exact test^34^ on overlapping genes in the top 100, 200,..., 5000 significant genes inferred from male and female samples separately. The results were shown in Supplementary Data S6. Interestingly, opposite patterns were identified for different drugs. For 11 (out of 14) drugs tested, the overlap becomes significant (at p-value ≤ 0.05) if more than 500 top genes are selected from male and female groups. The results suggest that male and female might share a lot commonality on the response process of these drugs. However, the p-values are not constantly significant for the remaining 3 drugs. More differences (between male and female) were found for PD-0332991, RAF265, and ZD-6474. We performed an in-depth literature mining on the targeted cancers of the 14 drugs used in this study and summarize the results in Supplementary Table 2. There is only one drug PD-0332991 targeted purely on gender specific cancers, i.e., breast and ovarian cancer. Interestingly, PD-0332991 is also one of the three drugs showing to have gender effect according to our regression model. The rest two drugs, i.e., RAF265 and ZD-6474, though not designed specifically for gender specific cancers, such as prostate or ovarian cancer, also have some sort of gender specific effect for part of their targeting cancers. For example, RAF265 is reported to inhibit the growth of human melanoma tumors^35^ and men have much worse melanoma survival than woman^36^. ZD6474 is an inhibitor of VEGFR-2, which is designed for controlling the growth of lung metastasis and pleural effusions in human non-small cell lung cancer^37^. There are also clinical evidence showing that ZD6474 has potential role in the targeted therapy of breast cancer^38^. Men and women are shown to be different in both formation and prognosis of lung and breast cancers^39^. We also selected 9 drugs and plot their overlap in DRA genes between male and female in Fig. 4b. In summary, the two genders share common mechanisms for cancer drug responses for many drugs. However, there might be two patterns of DRA genes between genders.

### Age effect of drug sensitivity

To investigate the impact of age in drug response mechanisms, we divided the samples evenly into two groups (namely young group and old group) by chronological age and repeated our analyses on these two groups. We listed the detailed information including sample sizes, numbers of significant DRA genes, and the age to separate the young and old for 14 drugs in Supplementary Table 3. As can be seen, the median age is 53 or 54 for all the drugs, which is not surprising since cancer is mainly an aging disease. The DRA genes show quite different patterns between young and old groups. Except for Irinotecan, RAD265, and ZD-6474, there are several to several hundred DRA genes for young group. However, the numbers of DRA genes for old group are close to zero for most drugs. Interestingly, there is only one significant DRA gene for 17-AAG in old group, however it is also identified as DRA for young group (with Fisher’s exact test p-value for overlap being 2.4 × 10^−3^). Similar scenario was observed for Topotecan (p-value 2.0 × 10^−3^). It is known that age has a huge effect on the sensitivity of drugs and most drugs are more sensitive to young people^40^. Our results show that this difference might due to loss of function of some genes for the elder. However, we are fully aware that the small sample size should contribute at least in partial to the difference.

### Drug-sensitivity associated gene modules and their key driver genes

We have considered the relationship between individual gene expression and cell line-drug sensitivity. However, the sensitivity of a drug might relate to many genes and their interactions, thus it will be helpful to view the relationship from network perspective. Similar to Staunton *et al.*^41^, for each drug we divided the cell lines into three groups, namely sensitive group, indeterminate group, and resistance group. Specifically, cell lines with sensitivity values at least 0.8 standard deviations greater than the mean for a drug were defined as resistant to the drug; those with 0.8 standard deviations below the mean were defined as sensitive; and cell lines with sensitivity values within a window of 1.6 standard deviations around the mean were considered as indeterminate and were eliminated from analysis.

To perform the differential network module analysis, we considered the biological modules defined by Gene Ontology^42^ and Kyoto Encyclopedia of Genes and Genomes^43^ (see Methods). It is of note that we also tried to define modules based on gene co-expression using WGCNA^14^. However, the modules obtained by sensitive and resistance group are not consistent and the sample sizes are also relatively small in each group. Thus, we adopted modules in GO and KEGG, which represent more general gene modules. Specifically, for each module, we first overlapped it with 20,069 protein coding genes (downloaded from http://www.genenames.org/cgi-bin/statistics on 5-15-2015) and removed modules with less than 30 genes. 2,846 GO terms and 211 KEGG pathways passed the filtering.

We then applied modular differential connectivity (MDC) analysis^13^ to quantify co-expression difference between the resistant and sensitive states. MDC calculates the ratio between the average connectivity for gene pairs in resistant group and that for gene pairs in sensitive group. It is a continuous measure ranging from 0 to infinity. A module with MDC larger than (less than) 1 gains (loses) connectivity when changing from sensitive state to resistance state. The significance of MDC is estimated by a permutation study on cell lines (see Methods). We listed the MDC and significance for each GO and KEGG term in Supplementary Data S7. For a better view, we also showed top GO terms (with FDR 0 and extreme MDC values) for drug 17-AAG in Table 3. The top modules gaining and losing connectivity include GO:0032371 regulation of sterol transport, hsa04721 Synaptic vesicle cycle, and GO:0017156 calcium ion-dependent exocytosis. Cancer resistance proteins (e.g., ABCG and PCRP) have been implicated in the transport of sterols^44^, and exocytosis is one of the known mechanism related to multi-drug resistance in cancers^45^. In addition, modules related to cell membrane, drug metabolic process, synaptic transport, and immune systems are also in the top of the list.

For an overview of the MDCs of each GO term and KEGG pathway in multiple drugs, we listed their rank for each drug in Supplementary Tables 8 and 9 respectively and further ordered them according to the sum of the ranks across all drugs. Interestingly, we find that the GO term GO 0000003_reproduction ranks first at 7 out of 15 drugs tested. It is known that the power in inhibiting cell growth and reproduction is highly associated with cancer drug sensitivity^27^. In addition, consistent with the function analysis for DRA genes, modules related to cell cycle (e.g., cell cycle checkpoint, G1/S transition of mitotic cell cycle, and mitotic cell cycle) rank at the top.

For each significant module, it is very important to identify up-stream genes or hub genes directly interact with most genes in the module. The so called key driver genes or hub genes hold the key for reducing drug resistance and improving drug efficacy. They are usually identified by a few computational methods based on gene regulatory networks or protein interaction networks, e.g., key driver analysis (KDA)^13^. We selected a few significant modules for KDA analysis^13^. Specifically, we first constructed a protein-protein interaction (PPI) network using the interaction confidence level 0.4 in STRING database (version 10). We then mapped the genes in selected differential modules into the PPI network, and then retrieved the sub-network containing all the genes in the 2-neighborhoods of the mapped genes. By 2-neighborhoods, we mean the genes having a distance less than or equal to 2 with the mapped genes. We then identified the key driver genes (hub genes) of the mapped modules genes in the sub-network using KDA analysis^13^. It is worth noting that usually the key driver genes are inferred using a directed regulatory network like Bayesian network, however the key driver genes inferred from un-directed PPI network are also proved to be quite useful^46^.

**Figure 5:**
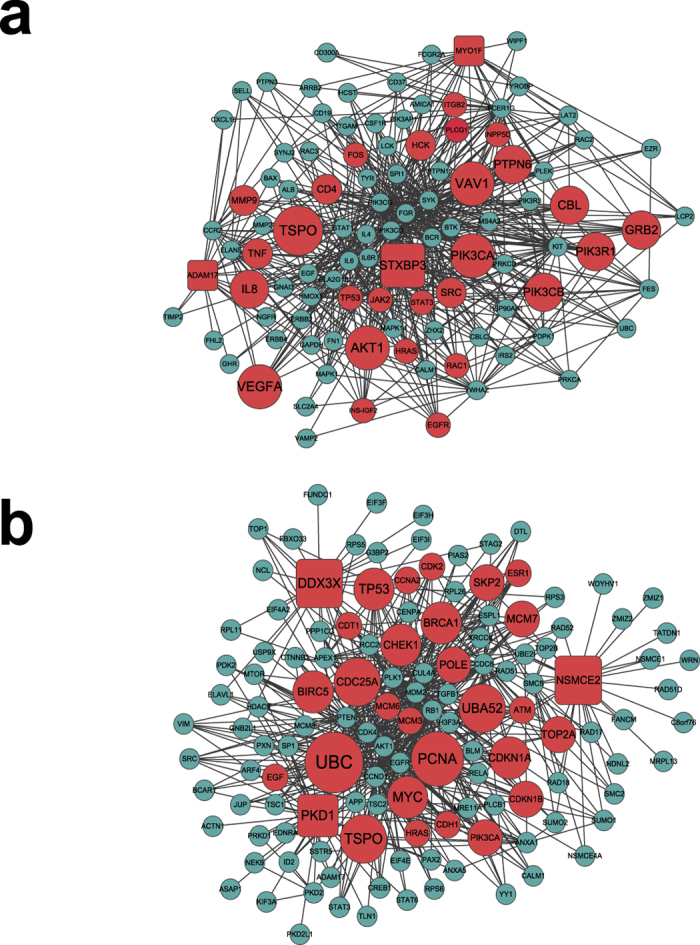
(**a**) Network view of genes in module GO:0002444 myeloid leukocyte mediated immunity and their neighboring genes for drug Paclitaxel, (**b**) Network view of genes in module GO:1901992 positive regulation of mitotic cell cycle phase transition and their neighboring genes for drug Topotecan. We use node shape to denote whether the node is in the module: (1) rectangle represents gene in module; (2) circle represents neighboring gene in the PPI network. We use color to denote whether the node is a key driver: (1) red represents key driver; (2) grey represents other gene.

We used module GO:0002444 myeloid leukocyte mediated immunity for drug Paclitaxel and module GO:1901992 positive regulation of mitotic cell cycle phase transition for drug Topotecan as two examples. The two networks were plotted in Fig. 5 using Cytoscape, in which the key drivers were in red and other genes in grey and the nodes with large size are more important. The top key driver genes for module GO:0002444 myeloid leukocyte mediated immunity are *VEGFA* and *TSPO*. *VEGFA* is a member of the PDGF/VEGF growth factor family, and has been identified to be related to cancer drug sensitivity in a couple of studies, e.g.^47^. Translocator protein (TSPO) is an 18 kDa high affinity cholesterol- and drug-binding protein found primarily in the outer mitochondrial membrane^48^. Interestingly, *TSPO* is also an important key driver gene for module GO:1901992 positive regulation of mitotic cell cycle phase transition. In addition, other genes including *UBC*, *PCNA*, and *TP53* were inferred to be top key drivers. In fact, somatic mutations of TP53 gene are one of the most frequent alterations in human cancers. TP53 mutations are also potential prognostic and predictive markers, as well as targets for pharmacological intervention^49^.

## Discussion

In this work, we presented a holistic view of the drug response related molecular markers including DRA genes and network modules for multiple drugs using the CCLE data. Both functional enrichment analysis on DRA genes and DRA network module analysis identified cell cycle as an important common function for multiple drug responses. It is known that many anticancer drugs functions by controlling the cell cycle which has been altered in human cancer^50^. In addition, our key driver analysis also infers *TSPO*, *TP53*, and many other immune or cell cycle related genes as key driver/hub genes of DRA modules. *TSPO* is important in binding small molecule drugs, cholesterol, and porphyrins^48^. The role of P53 in drug response has also been extensively studied^49^,^51^. For example, the p53 tumor suppressor are required to provoke cell apoptosis by DNA damage, which is crucial to the drugs inducing cancer cell apoptosis^51^. Destroying normal cell cycle, resisting apoptosis, and evading immune system are known hallmarks of cancer^52^. Interestingly, we also found the link between DRA genes and other cancer hallmarks. For example, GO:0000003 reproduction ranks first for 7 anticancer drugs among 2,846 GO terms (Supplementary Dataset S7), while the most significant hallmark of cancer is that the cancer cell can stimulate their own growth^52^. The modules related to other cancer hallmarks like genomic instability and inflammation are also rank as the top DRA modules. Thus the mechanisms of cancer drug sensitivity should be closely related to cancer hallmarks. In the future, it will also be interesting to study the potential of DRA genes or their co-expression patterns in predicting drug sensitivity by applying regression models.

Our study revealed two different patterns of drug response between two genders. The DRA genes were significantly overlapped for drugs targeting cancers that are common in both male and female, suggesting that there might be some fundamental drug response mechanisms conserved. However, very different drug response genes were identified for drugs only targeting gender specific cancers (e.g., breast cancer). Female sex has been shown to be a risk factor for clinically relevant adverse drug reactions. The female specific DRA genes might be the key to understand why female usually need more drug dosage even though they have small weight in general. Similarly, age is a main factor for drug response and many studies have shown that people in different age differ in drug response^11^. Our study showed much more DRA genes for young people than for old people, which suggests that some DRA genes might lose their functions with the increase of age.

We are fully aware that there might be a few limitations of this work. For example, the sample size is not large enough in especially the age and gender studies, which contributes at least partially to the differences in DRA genes identified. In addition, though we removed many confounding factors to reduce the potential false-positive DRA genes, there could be other factors unmeasured. An alternative way is to apply surrogate variable analysis^53^ to estimate these variables. However, this analysis usually estimate too many surrogate variables and it is not easy to determine a realistic number of variables. In addition, the estimated variables are not as reliable as the observed ones like gender and age. Thus, we prefer direct confounding factors as used in this study.

In addition, we used genes in GO and KEGG as modules to perform the differential module analysis due to the small sample sizes in sensitive and resistance groups. An alternative way is to define co-expression modules based on gene co-expression^13^, which is a more data-driven approach. It should also be better to construct Bayesian regulatory networks to infer the key driver genes, which considers the direction in regulation^13^. However, a large sample size is required for constructing robust co-expression and regulatory networks and we will try to integrate more samples in the future to perform such analyses.

Moreover, we would like to point out that linear model like the one we used has its limitation and may not be suitable for non-linear pattern discovery. We will test other non-linear models in the future. In the end, though our key driver genes have been supported by extensive literatures, some of them still need to be validated experimentally. In the future, it will be interesting to select a few less studied key driver genes for knock-out/knock-down study in specific cell lines.

## Methods

### Linear regression model of gene expression changes

For each drug, we assume gene expression is affected by drug sensitivity and other confounding factors including age, sex, batch, cancer and tissue types, and the top 3 genotype principal components denoting the race and other genetic factors (see GTEx study^54^). Similar to Yang *et al.*^55^ we model the gene expression change using the following linear regression formula:


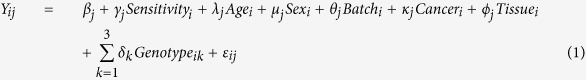


where, *Y*_*ij*_ is the expression level of gene *j* in sample *i*, *Sensitivity*_*i*_ is the drug sensitivity of sample *i*, *Age*_*i*_ denotes the age of sample *i*, *Sex*_*i*_ denotes the sex of sample *i*, *Batch*_*i*_ denotes the batch information of sample *i*, *Cancer*_*i*_ denotes the cancer type of sample *i*, *Tissue*_*i*_ denotes the tissue type of sample *i*, *Genotype*_*ik*_ denotes the *k*-th principle component value of the genotype profile for the *i*-th sample, *ε*_*ij*_ is the error term. In addition, *β*_*j*_ is the regression intercept, *γ*_*j*_ is the sensitivity regression coefficient, *λ*_*j*_ is the age regression coefficient, *μ*_*j*_ is the sex regression coefficient, *θ*_*j*_ is the batch regression coefficient, *κ*_*j*_ is the cancer type regression coefficient, *ϕ*_*j*_ is the tissue regression coefficient, *δ*_*k*_ is the regression coefficient for the *k*-th genotype PC. *Sex*_*i*_, *Batch*_*i*_, *Cancer*_*i*_, *Tissue*_*i*_ are factor variables.

If *γ*_*j*_ is significantly deviated from 0, gene *j* is considered to be drug sensitivity-associated. Gene *j* is positively associated with drug sensitivity if *γ*_*j*_ > 0 and negatively associated if *γ*_*j*_ < 0. We performed the false discovery rate (FDR) adjustment on the p-values using Benjamini Hochberg method and a FDR less than 0.1 is used as the significance threshold throughout the paper unless other thresholds are specified in special cases.

### Removal of confounding factors based on principle component analysis

Removing confounding factors is usually indispensable in revealing the true relationship between gene expression change and drug sensitivity. Besides sex, age, tissue type, batch, cancer type, major principle components (PCs) of genotype are also frequently used as confounding factors in gene expression analysis to boost true signal detection. Top genotype PCs usually covers the information of ethnicities and sub-population structures^54^. To ensure that over-fitting is not a big concern and the DRA genes we found are generally true, we performed permutation analysis. Specifically, we randomly permuted the sensitivity values of samples and repeated the test for 1,000 times. We then count the number of times that there are more DRA genes in permuted data than that in the original data and the average number of DRA genes in the permuted data, which serves as a background DRA genes detected by random.

### Function enrichment analysis

We performed functional enrichment analysis on the positively-regulated and negatively-regulated genes separately using David tools (http://david.abcc.ncifcrf.gov/summary.jsp). Benjamini score is used to control the false discovery rate and a gene set is considered to be DRA if the corresponding Benjamini score is less than or equal to 0.05.

### Gender and age effect of drug sensitivity

We separated the male and female samples and analyzed DRA gene expressions using the modified regression model. Then used one tailed Fisher’s exact test to calculate the significance of overlapping DRA genes between male and female on the top 100, 200,..., 5000 sensitivity-related genes. The sensitivity genes from male and female are considered to be significantly overlapped if the p-value of the test score is less than or equal to 0.05. Similarly, we separated the samples evenly into young and old group according to sample age and applied the modified regression model to data for separate groups respectively. As the sample size of each group is equal, we simply compared overlapping of DRA genes in each group using the Fisher’s exact test.

### Module Differential Connectivity (MDC) and Key Driver Analysis

We performed module differential connectivity analysis on functional modules defined by Gene Ontology^42^ and Kyoto Encyclopedia of Genes and Genomes (KEGG)^43^. The genes for each GO term and KEGG pathway were retrieved using R package “org.Hs.eg.db” and “KEGG.db”, respectively (on May 15, 2015). We only consider the modules with more than 30 protein coding genes.

For each module, we use the R package MDC^13^ to calculate the ratio of mean co-expression between the resistant and sensitive group. To evaluate the false discovery rate (FDR) for each module, we then used MDC to perform permutation tests for 50 times. Specifically, we randomly permuted the samples between the resistant and sensitive group. For each permutation, we calculated the ratio of mean co-expression between resistant and sensitive group. If MDC for original data is larger than (less than) 1, then the FDR is calculated as the number of times we have larger (smaller) MDC in the permuted group than in the original group.

The key drivers of the genes for each significant module were inferred by the package KDA^13^ on protein interaction network. Specifically, the protein-protein interaction (PPI) network is constructed using the interaction confidence level 0.4 in STRING database (version 10). We first mapped the genes in a significant module into the STRING PPI network. Let *A* denote the set of mapped genes. For each gene in the STRING PPI network, KDA retrieved all genes with distance less than or equal to 2 from the gene. Let *B* denotes the gene set. A hyper geometric test is used to calculate the significance of the enrichment between gene set *A* and *B*. We also permuted genes in the 2-neighbor of the gene to calculate the FDR and the genes with FDR less than 0.05 is considered as key driver genes. We plot the key driver genes and their connectivity in the PPI network using Cytoscape (http://www.cytoscape.org/).

## Additional Information

**How to cite this article**: Liu, X. *et al.* A systematic study on drug-response associated genes using baseline gene expressions of the Cancer Cell Line Encyclopedia. *Sci. Rep.* **6**, 22811; doi: 10.1038/srep22811 (2016).

## Supplementary Material

Supplementary Information

Supplementary Dataset S1

Supplementary Dataset S2

Supplementary Dataset S3

Supplementary Dataset S4

Supplementary Dataset S5

Supplementary Dataset S6

Supplementary Dataset S7

Supplementary Dataset S8

Supplementary Dataset S9

## Figures and Tables

**Table 1:** Number of sensitivity-associated genes in 24 drugs.

Drug	Sample Size	Gene^*a*^ (FDR ≤ 0.1)			Permutation^*b*^	
		Positive	Negative	Overall	Frequency	Average Number
17-AAG	323	253	180	433	0	0.12
AEW541	318	189	116	305	0	0.65
AZD0530	323			2	85	0.77
AZD6244	323	180	117	297	0	0.67
Erlotinib	318	332	120	452	0	3.61
Irinotecan	183	137	94	231	0	0.42
L-685458	316			4	134	2.28
LBW242	323			14	86	5.88
Lapatinib	323	768	458	1226	0	1.47
Nilotinib	260			80	49	25
Nutlin-3	323			4	0	2.47
PD-0325901	323	224	145	369	0	0.64
PD-0332991	271	21	9	30	1	0.55
PF2341066	323			13	61	3.76
PHA-665752	323			7	32	1.16
PLX4720	313			15	184	9.44
Paclitaxel	323	693	298	991	0	0.21
Panobinostat	316	290	306	596	0	0.28
RAF265	293	56	39	95	1	0.45
Sorafenib	318			163	1	15.36
TAE684	323	136	123	259	0	2.21
TKI258	323			10	155	6.12
Topotecan	323	363	182	545	0	0.40
ZD-6474	313	19	10	29	1	0.33

^*a*^Number of significant genes (at FDR ≤0.1) after adjusting gender, age, tissue, batch, cancer types, top three genotype principal components (PCs) as confounding factors. Columns “Positive”, “Negative”, and “Overall” list the number of positively-regulated, negatively-regulated and overall sensitivity-associated genes. ^*b*^Information of sensitivity-associated genes (at FDR ≤ 0.1) in 1,000 permutation runs. Column “Frequency” lists the frequencies of identifying equal or more significant genes in the permuted datasets than those in the original one for 24 drugs; Column “Average Number” lists the average number of sensitivity-associated genes in 1,000 permutation runs. The blank in the table represents the drugs with the small numbers of DRA genes (≤20) and higher false positive rates judged by the permutation test.

**Table 2:** Numbers of samples in sensitive and non-sensitive groups identified by expression pattern of DRA genes for 14 drugs and the significance of differences for sensitivity values between the two groups by the Student’s t-test.

Drug	NO. samples in Sensitive group	NO. samples in non-sensitive group	P-value for t-test
17-AAG	120	203	<2.2 × 10^−16^
AEW541	234	84	2.8 × 10^−11^
AZD6244	172	150	1.2 × 10^−15^
Erlotinib	51	267	2.0 × 10^−7^
Irinotecan	133	50	<2.2 × 10^−16^
Lapatinib	69	254	5.1 × 10^−10^
PD-0325901	221	102	<2.2 × 10^−16^
PD-0332991	234	37	2.6 × 10^−10^
Paclitaxel	72	250	7.3 × 10^−8^
Panobinostat	159	156	<2.2 × 10^−16^
RAF265	93	200	1.3 × 10^−7^
TAE684	109	214	2.1 × 10^−10^
Topotecan	154	168	<2.2 × 10^−16^
ZD-6474	191	122	1.6 × 10^−14^

**Table 3:** Top modules that gain or lose connectivity between sensitive and resistance groups for drug 17-AAG.

Module	MDC	Module	MDC
GO:0032371 regulation of sterol transport	25.54	hsa04721 Synaptic vesicle cycle	0.1
GO:0032374 regulation of cholesterol transport	25.54	GO:0017156 calcium ion-depend. exocytosis	0.13
hsa04610 Complement and coagulation cascades	16.74	GO:0016079 synaptic vesicle exocytosis	0.16
GO:0006953 acute-phase response	16.6	GO:0016486 peptide hormone processing	0.16
GO:0030195 negative regulation blood coagulation	14.53	GO:0048489 synaptic vesicle transport	0.17
GO:0072376 protein activation cascade	11.47	GO:0021954 central nervous syst. develop.	0.22
GO:0071827 plasma lipoprotein particle organization	10.35	GO:0000380 alternative mRNA splicing	0.23
GO:0097006 regula. plasma lipoprotein particle level	9.64	GO:0051899 membrane depolarization	0.23
GO:0017144 drug metabolic process	5.87	GO:0042093 T-helper cell differentiation	0.31
GO:0002526 acute inflammatory response	5.4	GO:0000082 G1/S transition of mitotic cell cycle	0.73
