# Supplementary material for: A systematic study on drug-response associated genes using baseline gene expressions of the Cancer Cell Line Encyclopedia
Source: Sci Rep. 2016 Mar 10;6:22811. doi: 10.1038/srep22811 (PMC4785360; doi:10.1038/srep22811)
Supplement: Supplementary Information [file srep22811-s1.doc]

# **Supplementary: A systematic study on drug-response associated genes using baseline gene expressions of the Cancer Cell Line Encyclopedia**

Running Title: Systems study on drug-response associated genes

**Xiaoming Liu^1^, Jiasheng Yang^2^, Yi Zhang^3^, Yun Fang^1^, Fayou Wang^1^, Jun Wang^1^, Xiaoqi Zheng^1,*^, and Jialiang Yang^3,5^,***

^1^ Department of Mathematics, Shanghai Normal University, Shanghai 200234, P. R. China

^2^ College of Veterinary Medicine, Yangzhou University, Jiangsu 225009, P. R. China

^3^ Department of Mathematics, Hebei University of Science and Technology, Shijiazhuang, Hebei 050018, P. R. China

^4^ Department of Civil and Environmental Engineering, National University of Singapore, Singapore 117576, Singapore

^5^ Department of Genetics and Genomic Sciences, Icahn School of Medicine at Mount Sinai, New York, NY 10029, USA

*Correspondence author:

*Jialiang Yang. Department of Mathematics, Hebei University of Science and Technology, Shijiazhuang, Hebei 050018, P. R. China.*

*Email:* [*jialiang.yang@mssm.edu*](mailto:jialiang.yang@mssm.edu)*.*

*Xiaoqi Zheng. Department of Mathematics, Shanghai Normal University, Shanghai 200234, P. R. China. Email:* [*xqzheng@shnu.edu.cn*](mailto:xqzheng@shnu.edu.cn)*.*

# Supplementary Figures

**Fig. S1 -** Expression patterns of DRA genes for 13 drugs: (a) PD-0325901, (b) PD-0332991, (c) Paclitaxel, (d) Panobinostat, (e) RAF265, (f) AEW541, (g) TAE684, (h) Topotecan, (i) ZD−6474, (j) AZD6244, (k) Erlotinib, (l) Irinotecan, and (m) Lapatinib

**
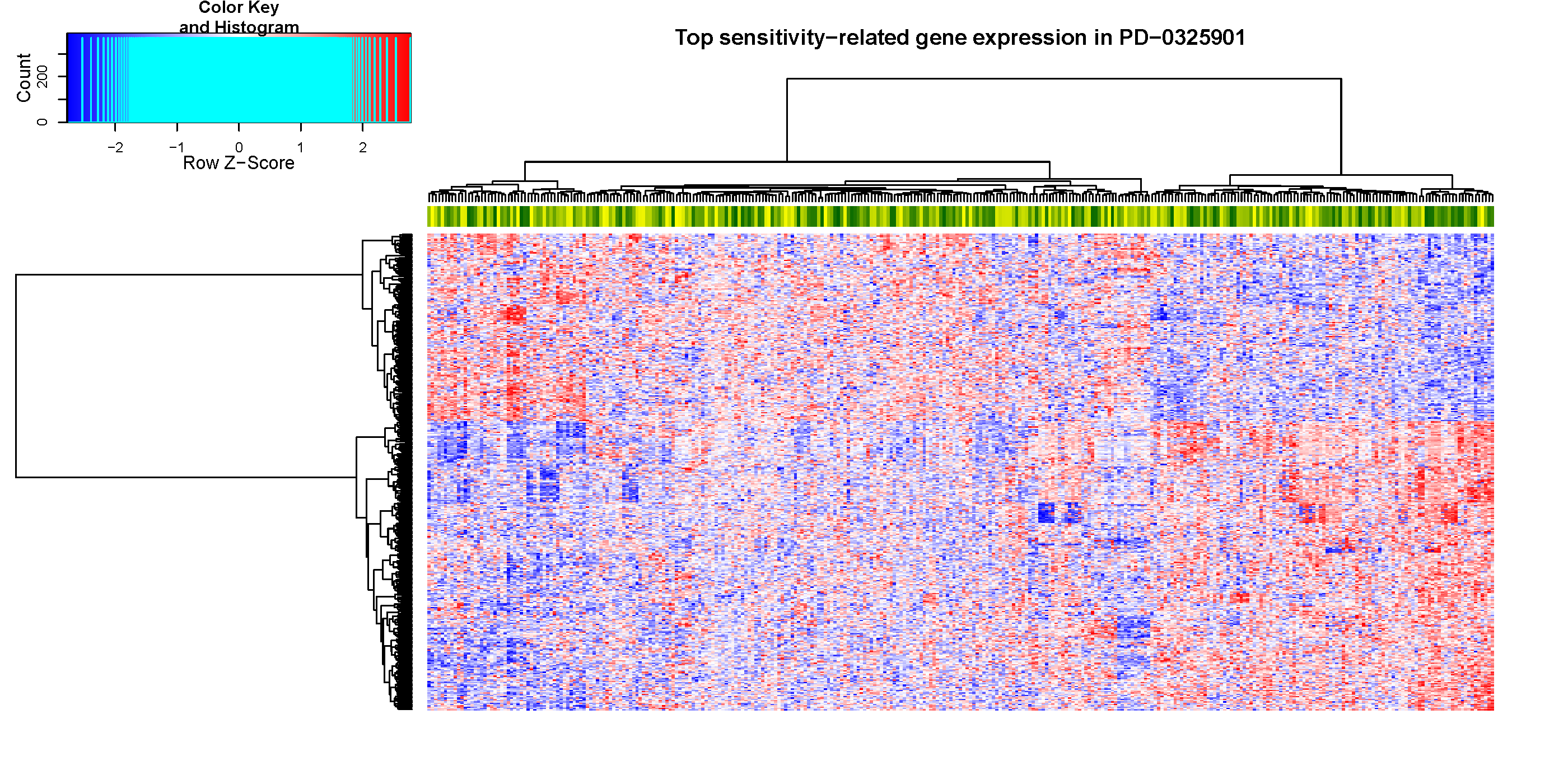
**

**(a)**

**
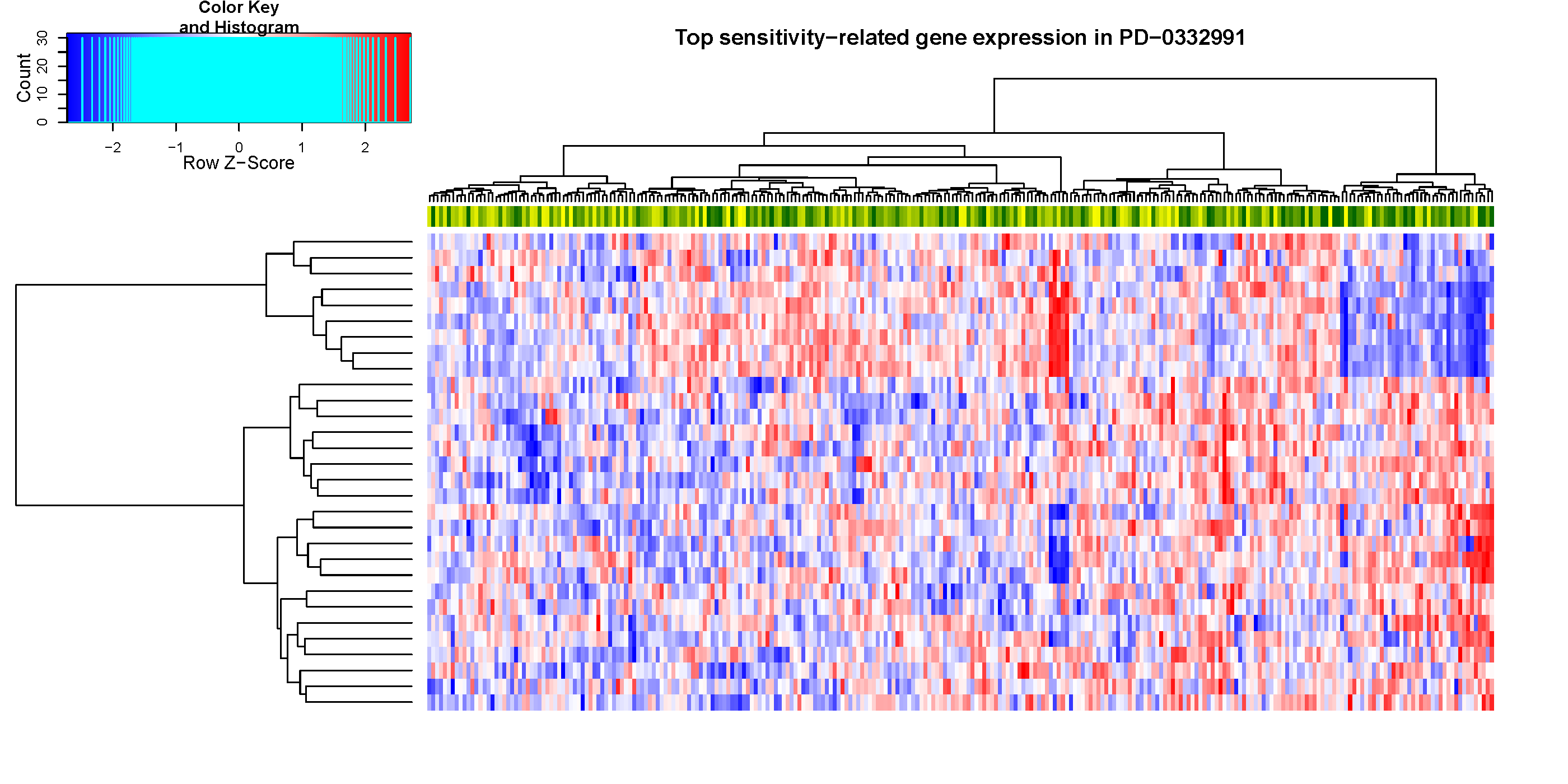
**

**(b)**

**
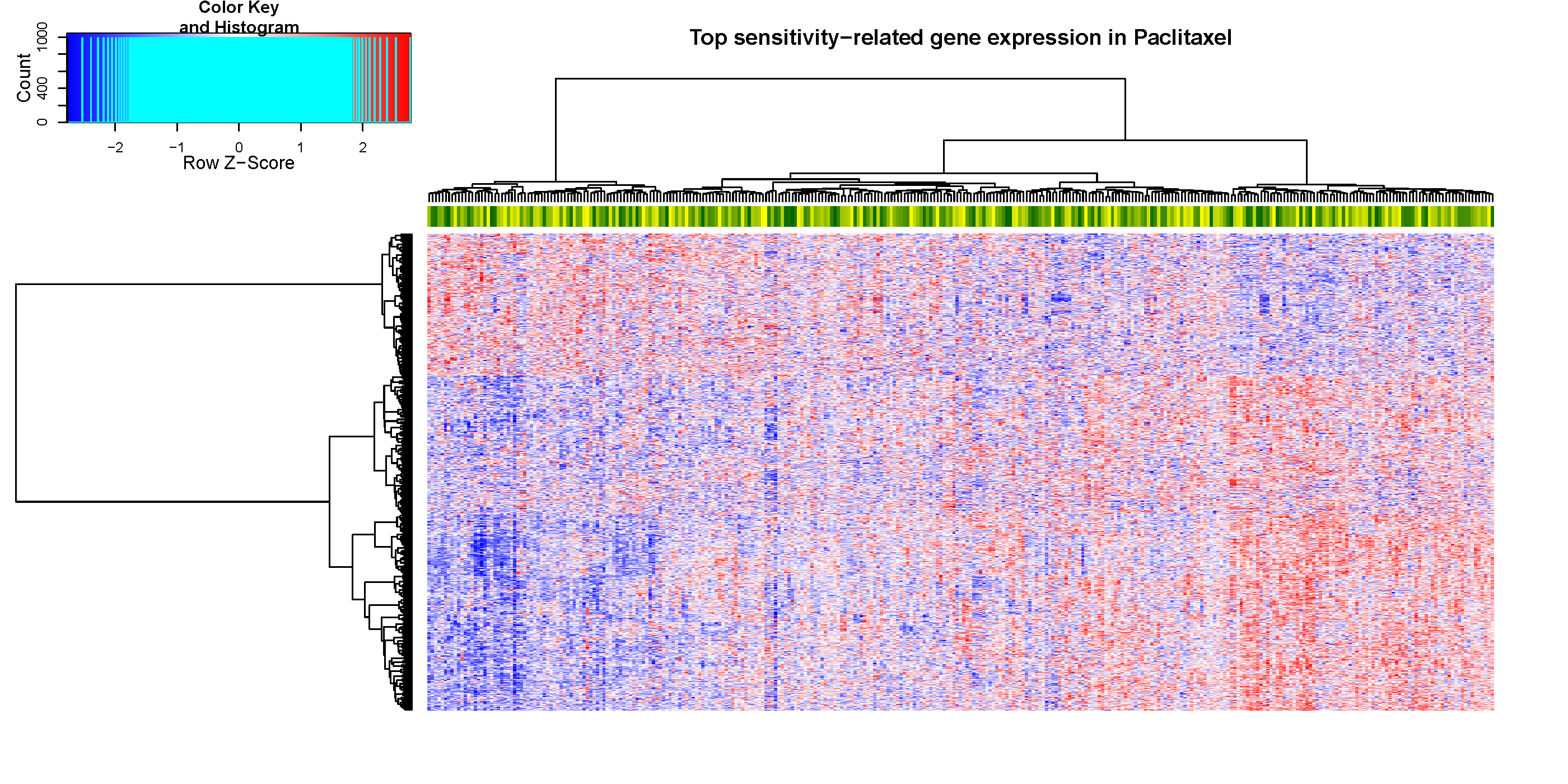
**

**(c)**

**
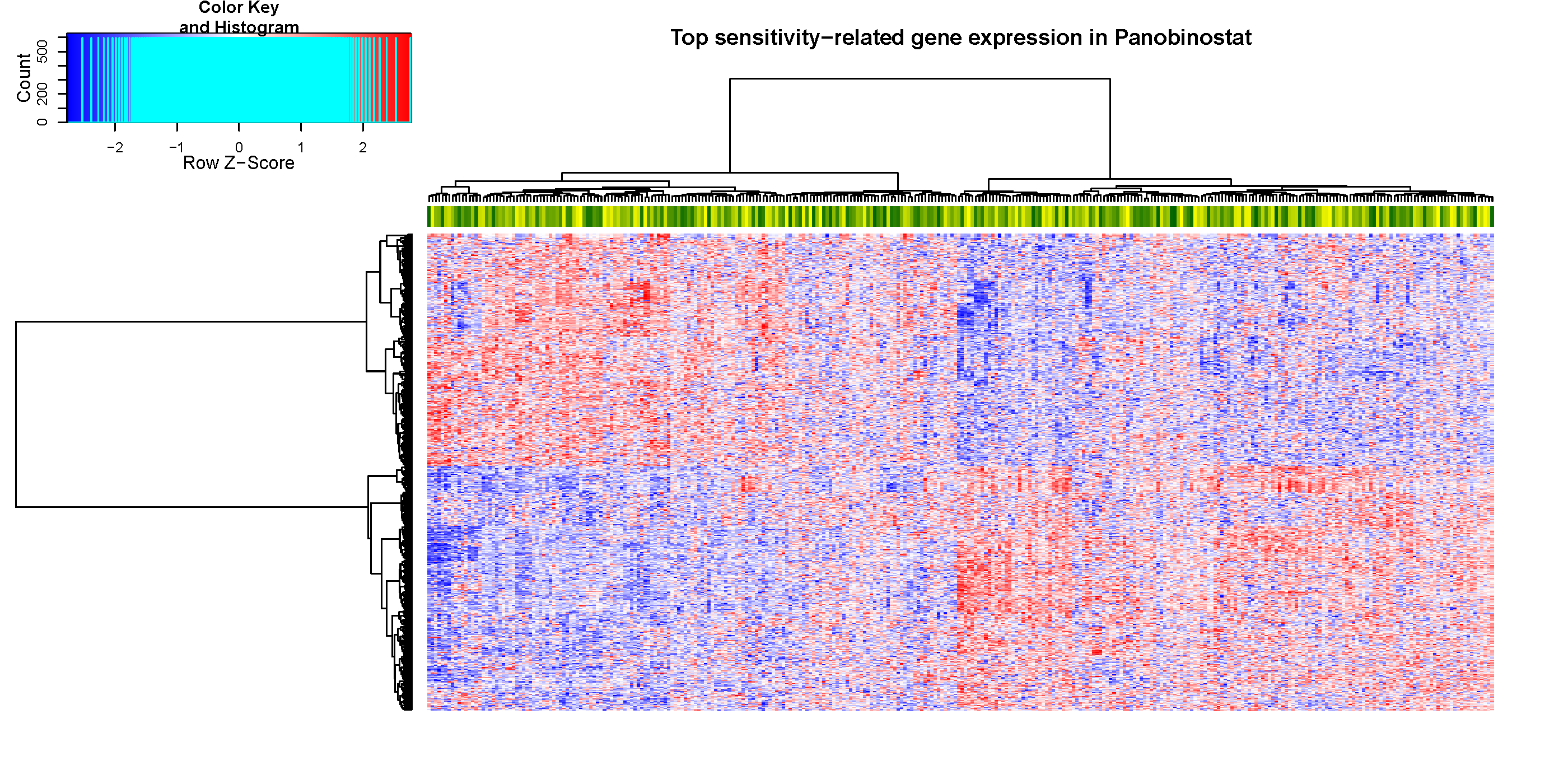
**

**(d)**

**
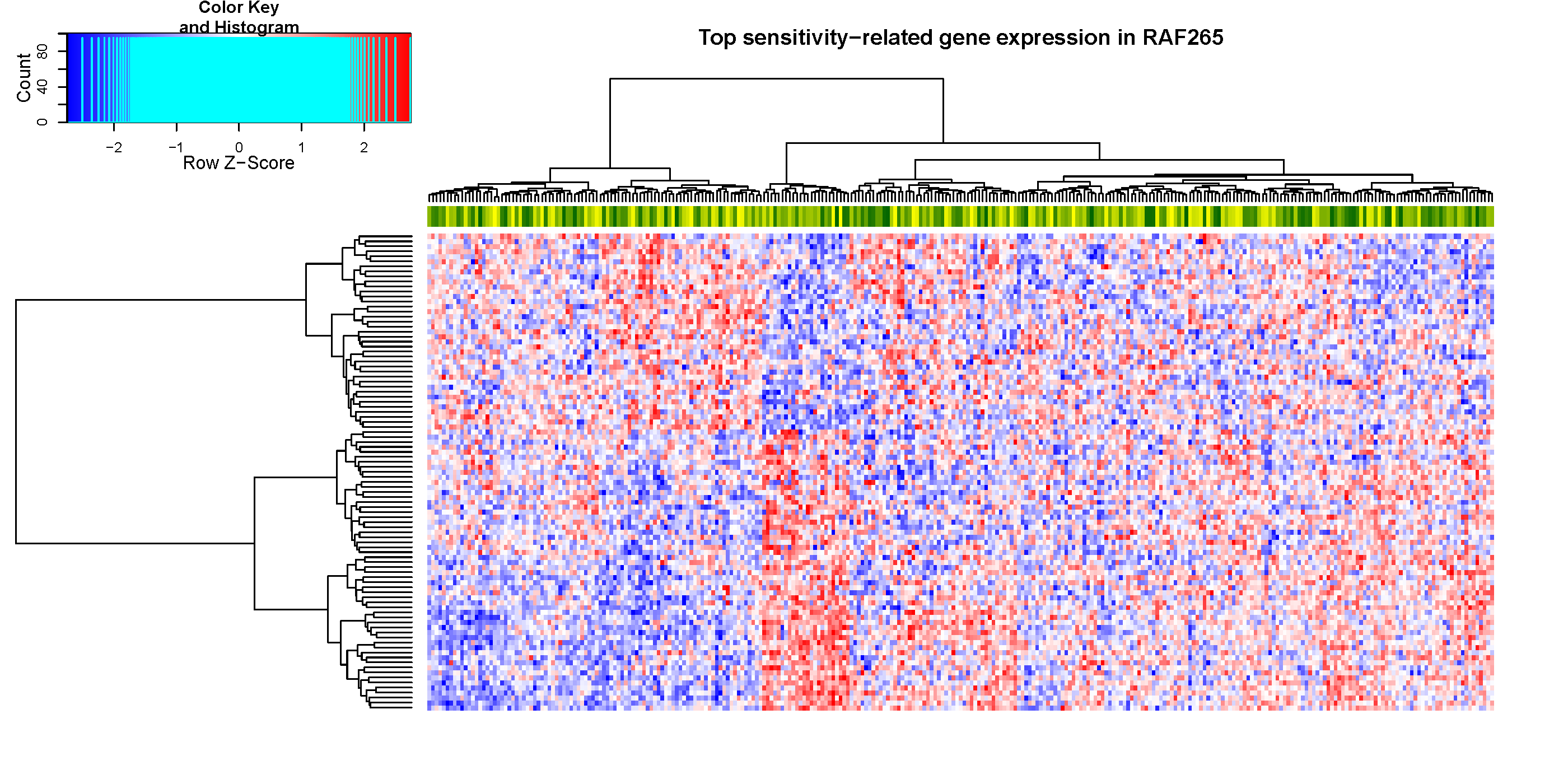
**

**(e)**

**
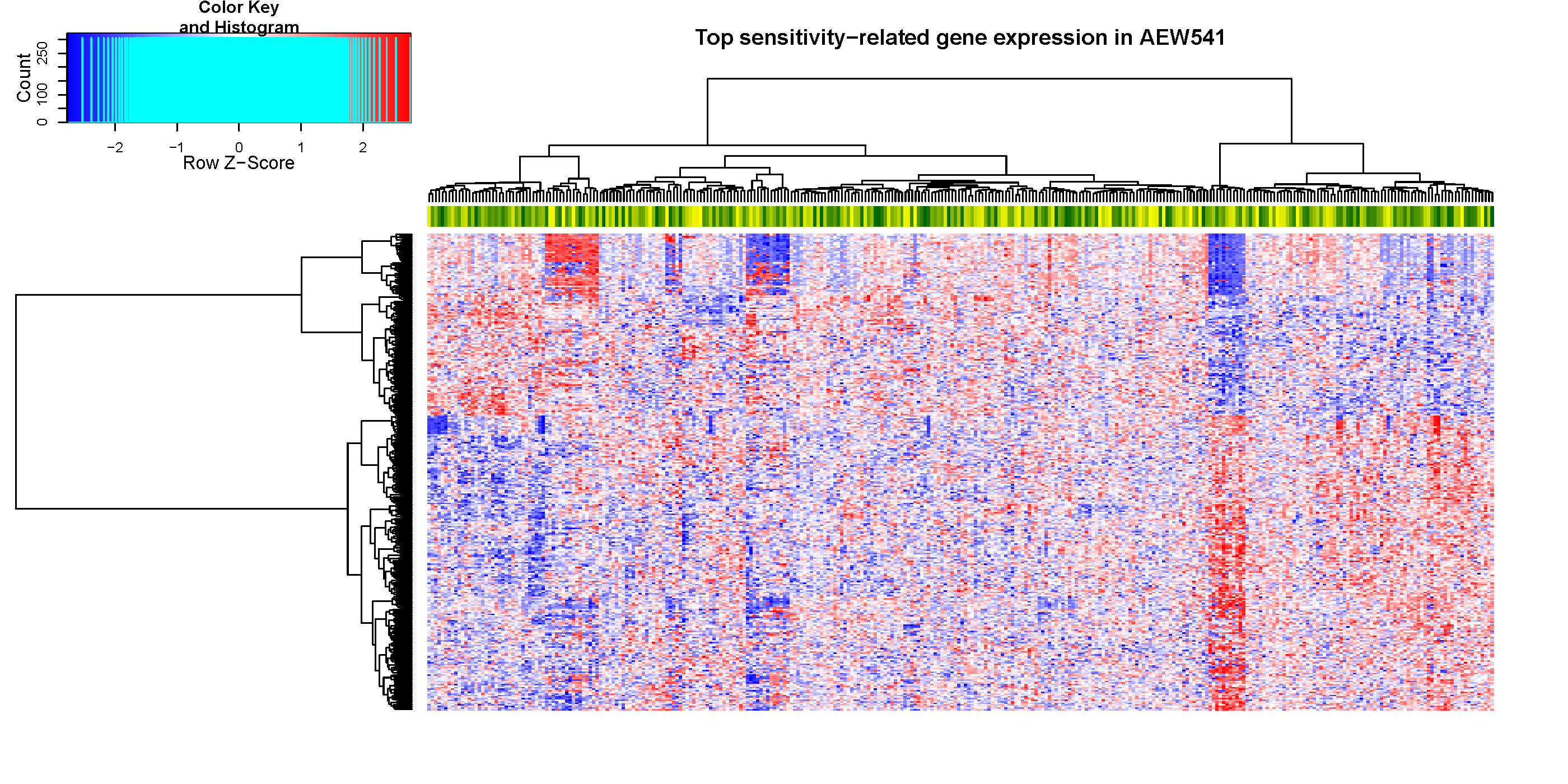
**

**(f)**

**
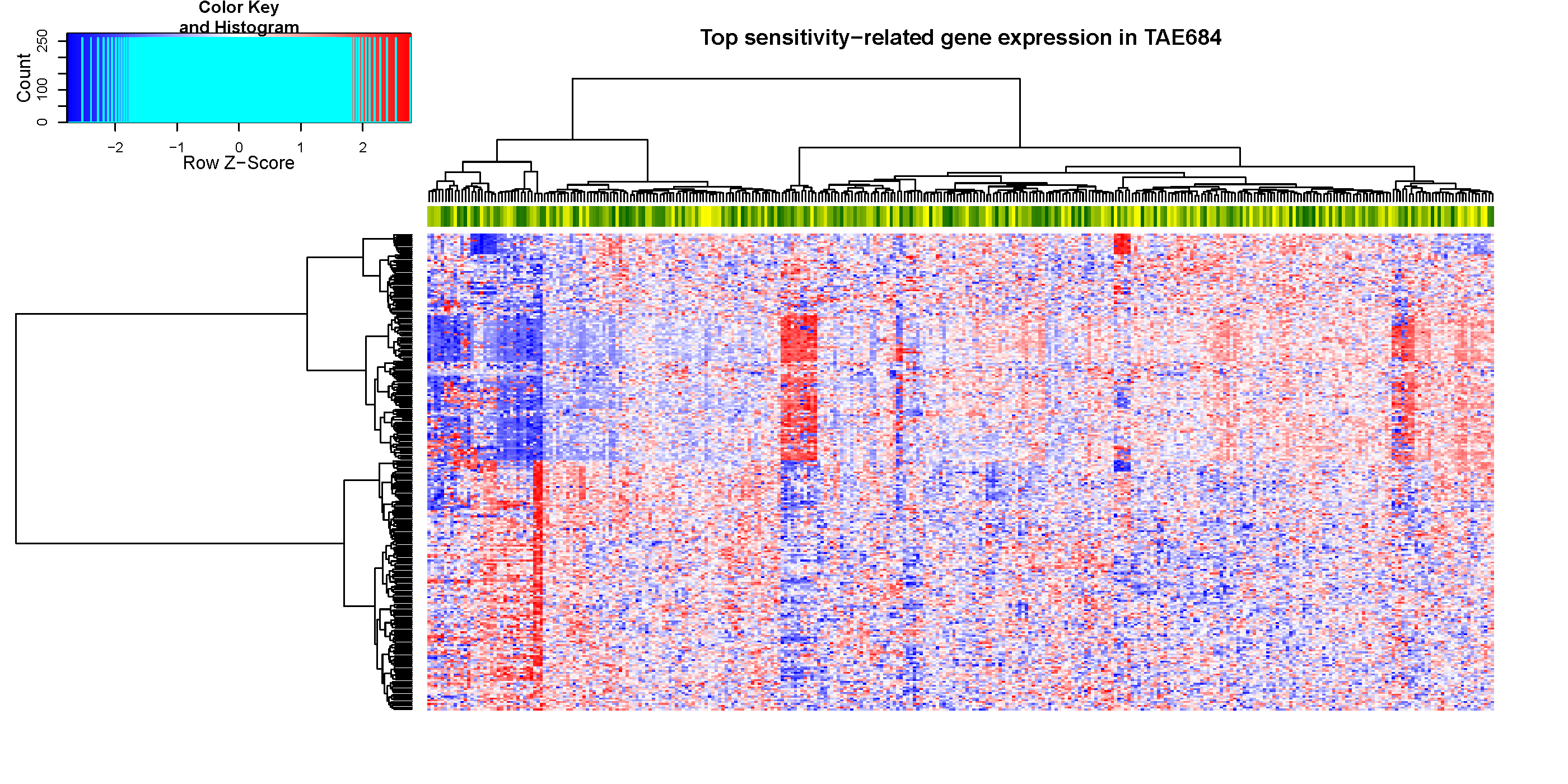
**

**(g)**

**
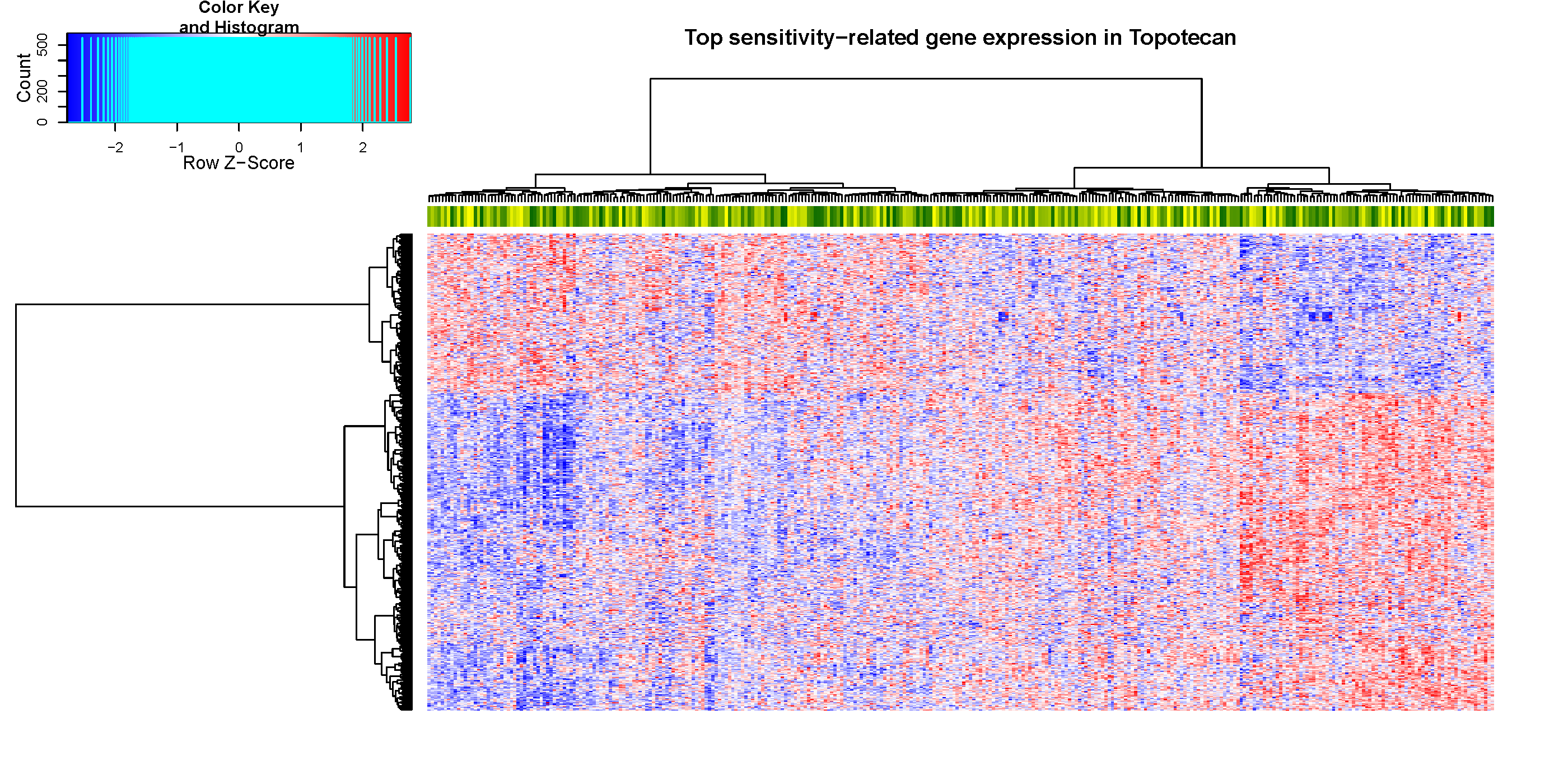
**

**(h)**


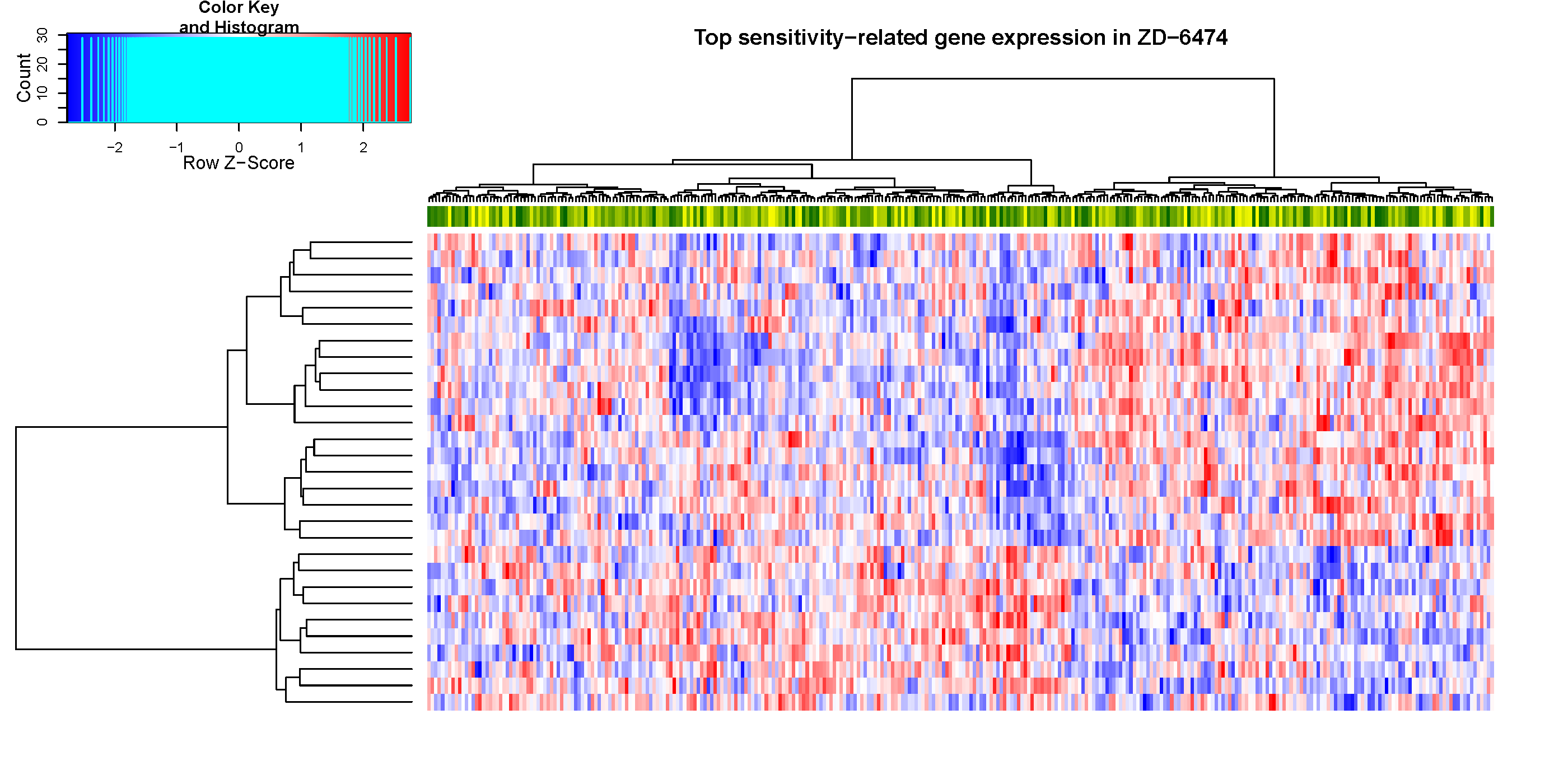


**(i)**


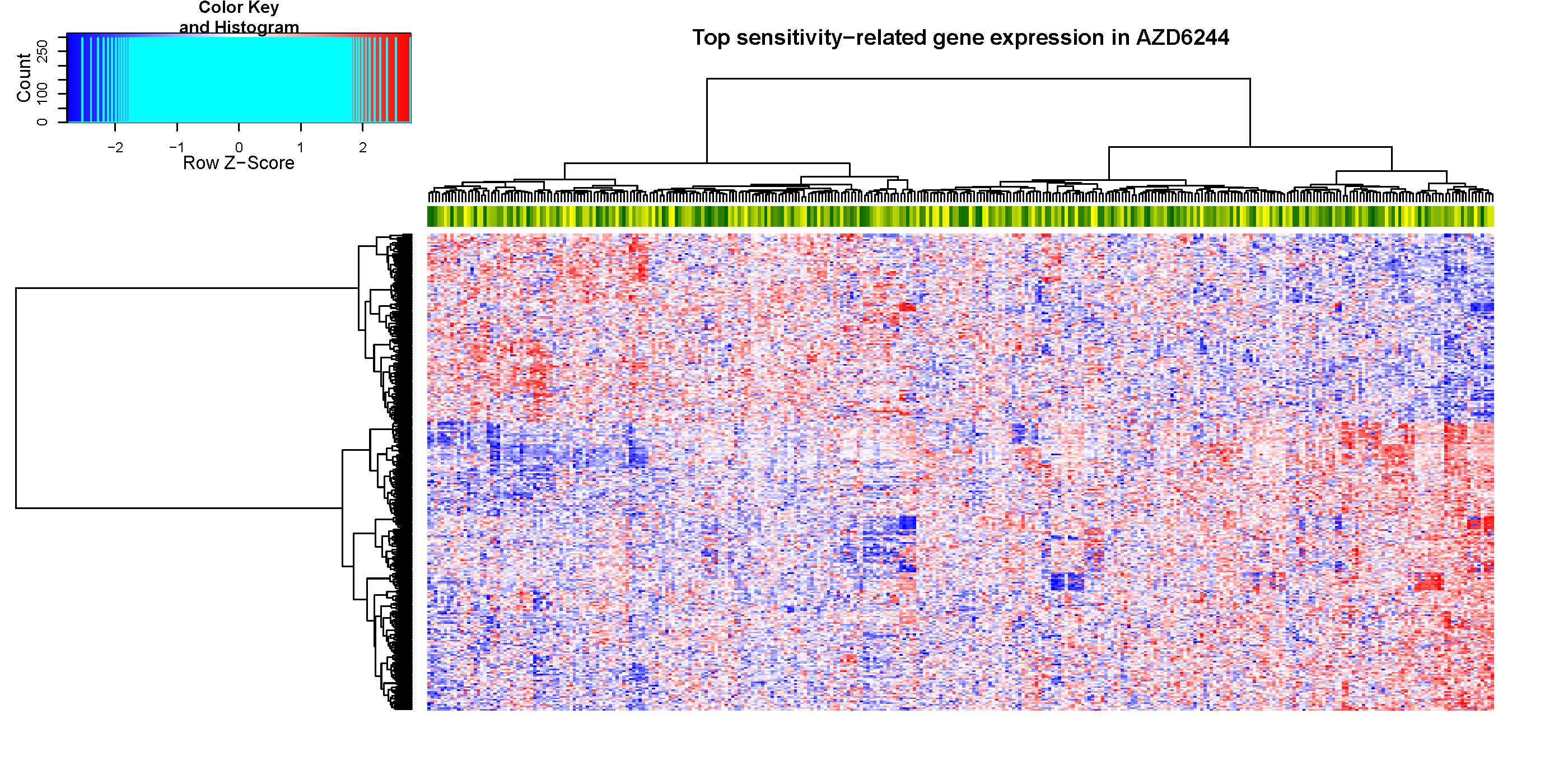


**(j)**


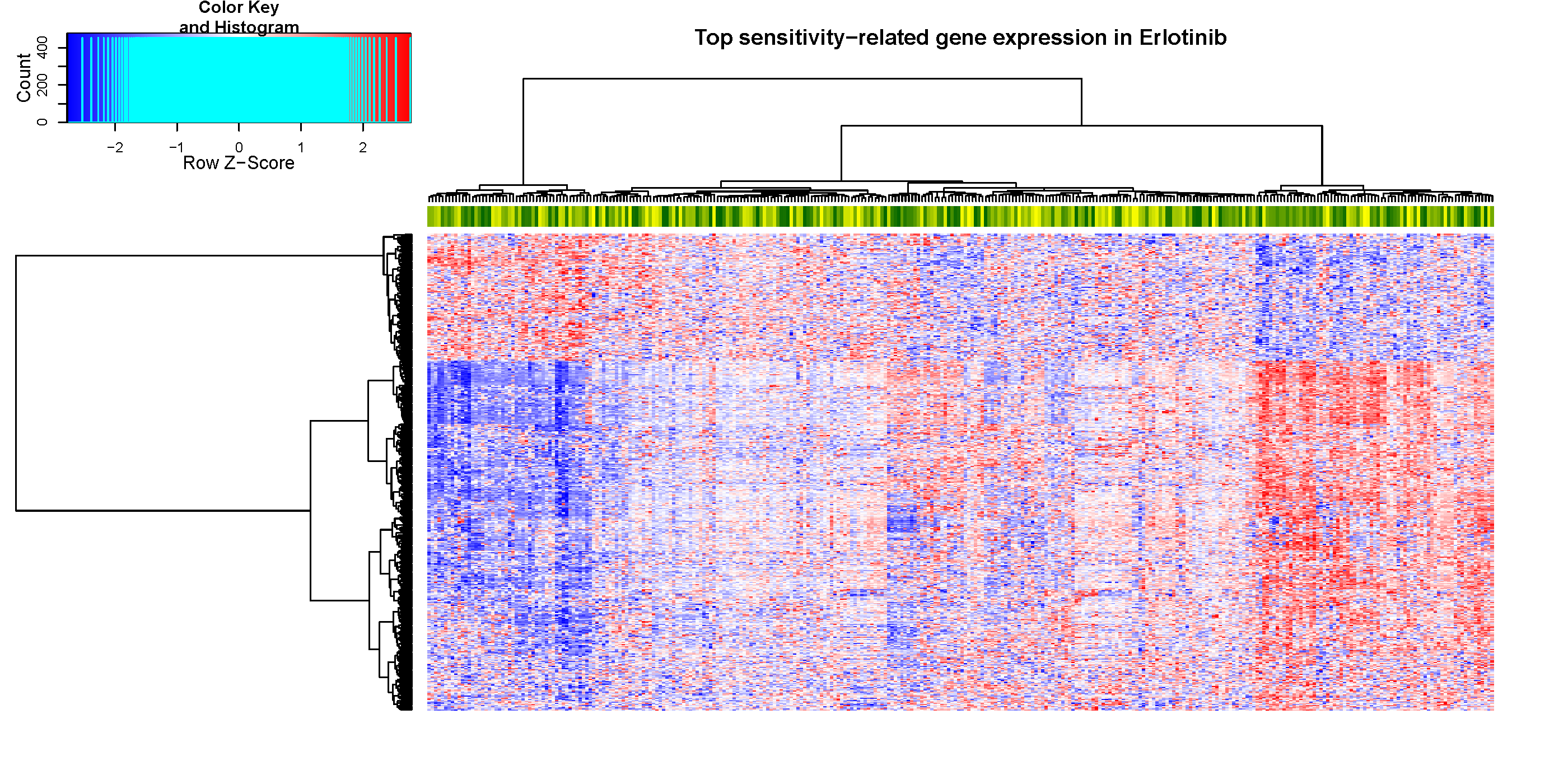


**(k)**


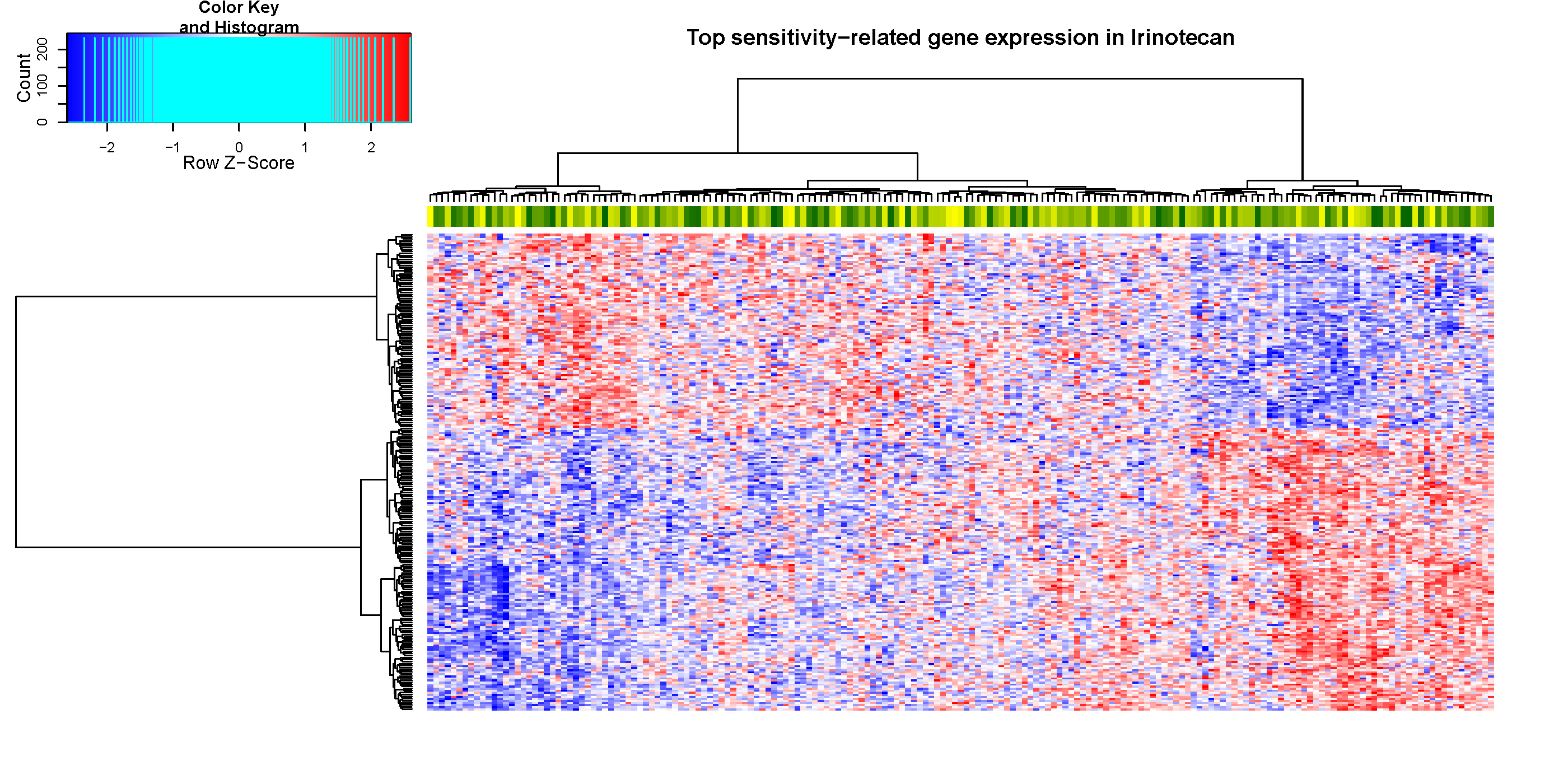


**(l)**


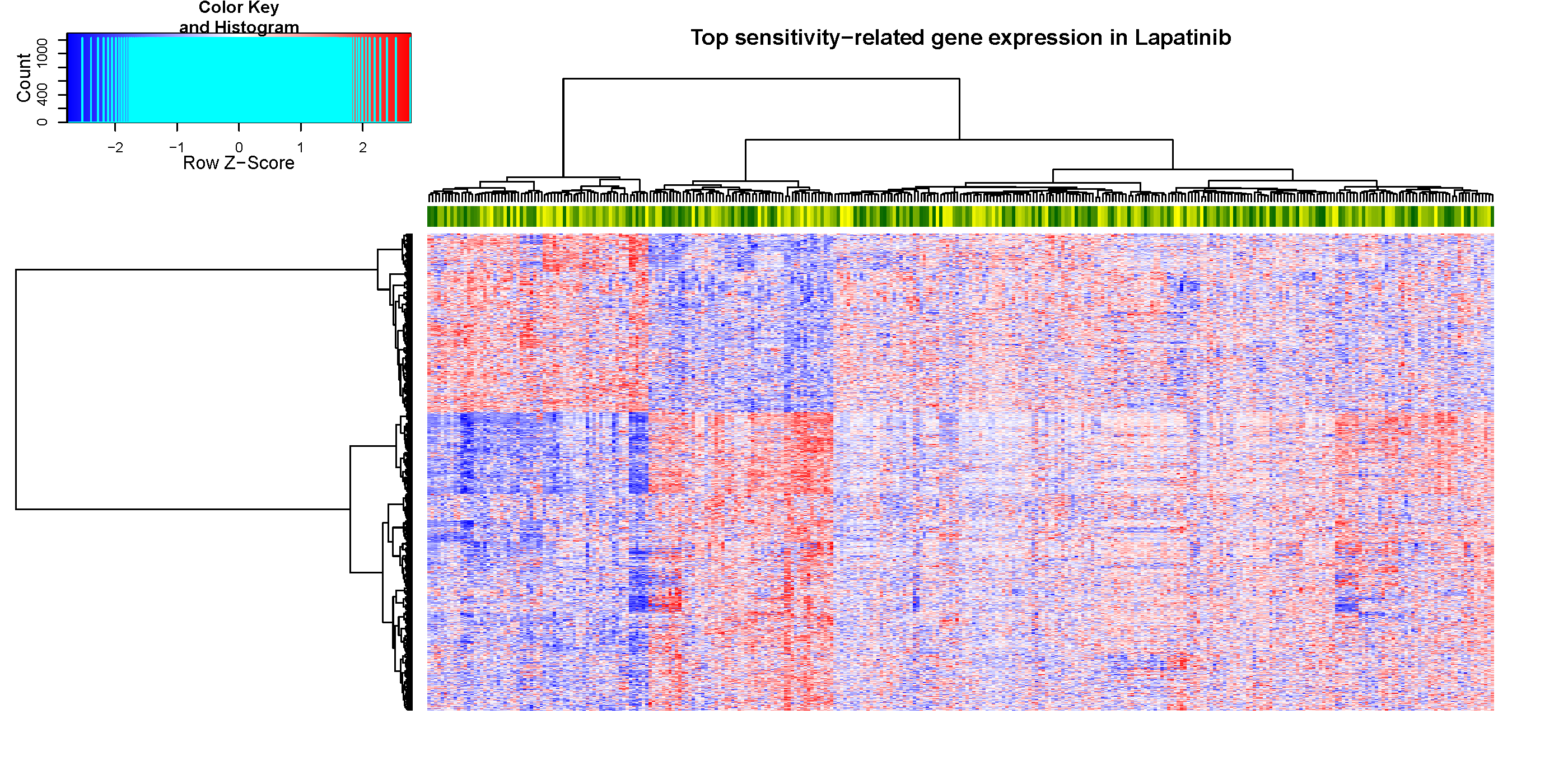


**(m)**

Rows indicate genes and columns indicate samples, Heat map colors represent normalized gene expression values with blue for low expression and red for high expression. X-axis represents sensitivity and Y-axis represents gene expression level. Pearson-R value in the title represents the Pearson correlation coefficient between gene expression and sensitivity across all samples.

# Supplementary Tables

**Table S1 – Number of samples in Male and Female**

| **Drug** | **Male** | **Female** | **Drug** | **Male** | **Female** |
| --- | --- | --- | --- | --- | --- |
| 17-AAG | 181 | 142 | PD-0332991 | 152 | 119 |
| AEW541 | 176 | 142 | Paclitaxel | 181 | 142 |
| AZD6244 | 181 | 142 | Panobinostat | 176 | 140 |
| Erlotinib | 176 | 142 | RAF265 | 169 | 124 |
| Irinotecan | 100 | 83 | TAE684 | 181 | 142 |
| Lapatinib | 181 | 142 | Topotecan | 181 | 142 |
| PD-0325901 | 181 | 142 | ZD-6474 | 176 | 137 |

**Table S2-Targeted cancers of 14 drugs in this study**

| **Drugs** | **Targets** |
| --- | --- |
| 17-AAG | colon and breast cancer, kidney tumors |
| AEW541 | biliary tract cancer |
| AZD6244 | [non-small cell lung cancer](https://en.wikipedia.org/wiki/Non-small_cell_lung_cancer) |
| Erlotinib | [non-small cell lung cancer](https://en.wikipedia.org/wiki/Non-small_cell_lung_cancer), [pancreatic cancer](https://en.wikipedia.org/wiki/Pancreatic_cancer) |
| Irinotecan | colon cancer |
| Lapatinib | breast cancer, gastric cancer |
| PD-0325901 | papillary thyroid carcinoma cells |
| PD-0332991 | breast, ovarian cancer |
| Paclitaxel | [lung](http://en.wikipedia.org/wiki/Lung_cancer), [ovarian](http://en.wikipedia.org/wiki/Ovarian_cancer), [breast](http://en.wikipedia.org/wiki/Breast_cancer), head and neck cancers |
| Panobinostat | T cell lymphoma |
| RAF265 | melanoma tumors |
| TAE684 | neuroblastoma lines |
| Topotecan | ovarian cancer, lung cancer |
| ZD-6474 | [non-small cell lung cancer](https://en.wikipedia.org/wiki/Non-small_cell_lung_cancer), breast cancer |

**Table S3-Number of sensitivity-associated genes using young and old samples separately**

| **Drugs** | **Young** | | **Old** | | **Overlap** | **Separation**  **Age** |
| --- | --- | --- | --- | --- | --- | --- |
|  | **Sample Size** | **Genes**  **(FDR <0.05)** | **Sample**  **Size** | **Genes**  **(FDR<0.05)** |  |  |
| 17-AAG | 161 | 48 | 162 | 1 | 1 | 54 |
| AEW541 | 159 | 76 | 159 | 0 | 0 | 54 |
| AZD6244 | 161 | 7 | 162 | 3 | 0 | 54 |
| Erlotinib | 159 | 23 | 159 | 1 | 0 | 54 |
| Irinotecan | 91 | 0 | 92 | 4 | 0 | 54 |
| Lapatinib | 161 | 535 | 162 | 0 | 0 | 54 |
| PD-0325901 | 161 | 6 | 162 | 0 | 0 | 54 |
| PD-0332991 | 135 | 4 | 136 | 12 | 0 | 53 |
| Paclitaxel | 161 | 300 | 162 | 3 | 0 | 54 |
| Panobinostat | 158 | 317 | 158 | 0 | 0 | 54 |
| RAF265 | 146 | 0 | 147 | 0 | 0 | 54 |
| TAE684 | 161 | 280 | 162 | 0 | 0 | 54 |
| Topotecan | 161 | 41 | 162 | 1 | 1 | 54 |
| ZD-6474 | 156 | 0 | 157 | 0 | 0 | 53 |

Number of significant genes (at FDR < 0.05) of the young group.

Number of significant genes (at FDR < 0.05) of the old group.

The Age to separate the young and old group.

# Supplementary Datasets

**Dataset S1**. Age, gender, tissue, batch, cancer type information for samples in 24 drugs

**Dataset S2***.* Sensitivity coefficients, p-values, and FDR for 20069 genes in 24 drugs

**Dataset S3**. Overlap of DRA genes among 14 drugs

**Dataset S4**. Overlapping of GO terms significantly enriched in DRA genes for 11 drugs

**Dataset S5**. Effect of sample size in identifying DRA genes for 14 drugs

**Dataset S6**. P-value of Fisher’s exact test between male and female

**Dataset S7**. The module differential analysis results for 15 drugs

**Dataset S8**. Ranking of modules defined by GO terms according to their association to sensitivity of 15 drugs

**Dataset S9**. Ranking of modules defined by KEGG pathways according to their association to sensitivity of 15 drugs
